# Individual variability in patterns and dynamics of fecal gluten immunogenic peptides excretion after low gluten intake

**DOI:** 10.1007/s00394-021-02765-z

**Published:** 2022-01-07

**Authors:** Laura Coto, Carolina Sousa, Angel Cebolla

**Affiliations:** 1 Biomedal S.L., Polígono Industrial Parque Plata, Calle Calzada Romana, 40, 41900 Camas, Sevilla Spain; 2 Human Nutrition and Food Science Doctoral Program, University of Granada, 18011 Granada, Spain; 3 Department of Microbiology and Parasitology, Faculty of Pharmacy, University of Seville, 41012 Seville, Spain

**Keywords:** Gluten immunogenic peptides, Gluten metabolism, Gluten detection feces, Gluten-free diet monitoring, Celiac disease

## Abstract

**Purpose:**

Determination of Gluten Immunogenic Peptides (GIP) in feces is a direct tool for gluten exposure detection. The sensitivity of GIP detection methods for cases of unintentional low gluten intakes is unknown. We studied the interindividual variability in the kinetic of excretion under homogeneously controlled dietary conditions, and the sensitivity of fecal GIP tests after low amounts of punctual gluten ingestions.

**Methods:**

Participants (*n* = 20) followed the same gluten-free menu for 12 days in which two separated doses of gluten (50 mg and 2 g) were ingested and all the depositions were collected. GIP from stool samples were analyzed by ELISA and lateral flow immunoassay (LFIA) tests.

**Results:**

Most participants had detectable GIP after 50 mg and 2 g gluten ingestions using ELISA test (72.2% and 95%, respectively), whereas the LFIA test showed less sensitivity (22.2% and 80%, respectively). GIP were detected at higher either frequency or concentration in the range of 12–36 h after 50 mg intake, and 12–84 h after 2 g consumption. Considering this period, diagnostic sensitivity of GIP detection after a single 50 mg ingestion may be significatively increased analyzing three stool samples per individual. High variability among participants was found in the time and amount of GIP excretion; however, some individuals showed common patterns for both gluten intakes.

**Conclusion:**

Sporadic gluten exposure detection may require several fecal samples to achieve level of sensitivity above 90%. Interindividual variability in the dynamic of GIP excretion may suggest patterns of gluten metabolism.

## Introduction

Celiac disease (CD) is a chronic, multiorgan autoimmune disease that occurs in genetically predisposed individuals with well-known genetic components such as human leukocyte antigen (HLA-DQ2 and HLA-DQ8), an auto-antigen (tissue trans-glutaminase), and gluten [1]. Additional environmental contributors have been suggested to be involved in its development, such as infections, imbalanced intestinal microbiota, and increased intestinal permeability [1–3]. The pathogenesis of CD involves the passage of gluten immunogenic peptides (GIP) through the intestinal barrier into the lamina propria via the trans- or paracellular route, with consequent activation of both adaptive and innate immune responses [4–7]. Reportedly, the α-gliadin 33-mer peptide is one of the most dominant immunogenic peptides, which contains three–six different potential T-cell epitopes of CD [8]. Three other gluten peptides are shown to produce most of the immunogenic responses observed in patients with CD [9].

Gluten is a water-insoluble polymorphic mixture of storage proteins, which are mainly categorized as alcohol-soluble prolamins and the alcohol-insoluble glutelins. Cereal grains such as wheat, rye, and barley contain high quantities of gluten, whereas oats show much lower content [10]. Prolamins impart specific functional properties such as viscoelasticity to food product; therefore, these cereals are used in a broad range of foodstuffs [11]. Prolamins are structurally characterized by unique repetitive amino acid sequences, rich in glutamine and proline; therefore, they are not easily digested by gastric and pancreatic enzymes [10].

Dietary gluten proteins are partially hydrolyzed in the stomach over a few minutes to 2–4 h, depending on the diet. Proteolytic degradation primarily occurs in the small intestine as a result of pancreatic enzyme activity, which cleaves polypeptides into small peptides and amino acids that are absorbed by transport systems [12, 13]. Previously, it was assumed that only di- or tripeptides are absorbed thorough the intestines; however, studies have shown that longer-chain gluten peptides resistant to digestion can enter the portal circulation, undergo filtration by the kidneys, and get excreted in urine [14]. Undigested gluten proteins and large peptides that remain intact in the small intestine may serve as substrate for local microbiota able to hydrolyze gluten [15]. The residual undigested peptides finally enter the large intestine, which contains a high density of living bacteria, and are further hydrolyzed over at least 10 h or even several days. The length and the activity of the hydrolytic process are largely dependent on the gut microbiota and the nature of the protein and the food matrix. Food components, including gluten proteins, that are not digested by enzymes and intestinal microbiota undergo fecal elimination [12, 13, 16, 17].

Strict lifelong compliance with a gluten-free diet (GFD) is the only treatment currently available for patients with CD, which implies avoidance of all gluten-containing foods and close attention to cross-contamination [18]. However, complete exclusion of dietary gluten is difficult in real-world practice because of the ubiquitous nature of gluten, social aspects and socioeconomic factors [19–21]. Consequently, GFD compliance in patients with CD was reported to be between 12 and 90% in adults [22–24] and between 23 and 98% in children [25], being asymptomatic patients more susceptible to regular gluten ingestion [22, 23]. A recent study reported that frequent gluten consumption showed by excreted GIP was associated with histological lesions, which may lead to future complications as a result of their condition [23].

Currently, a safe threshold of daily gluten intake among those with CD is unavailable, and the immune response to gluten significantly varies among this patient population [26]. Catassi et al. [27] concluded that the daily ingestion of contaminating gluten should be < 50 mg, based on histopathological findings observed in patients who received this dose. Additionally, other authors reported a gluten intake below 30 mg to avoid intestinal mucosal abnormalities [28].

Estimation of fecal and urinary GIP is the most direct method to monitor gluten ingestion [14, 17, 29]. In addition to control the adherence to a GFD, this method may also enable direct and quantitative assessment of gluten exposure, which refers to unintended low gluten intake. Information about the specific time, amount of ingested gluten and expected sensitivity for occasional gluten exposure may be important to design either the frequency of testing or the protocols to assess GFD compliance in patients with CD. Despite other authors have reported information about the process of GIP elimination in feces [17, 30–32], no data are available to date regarding the dynamic of GIP excretion, the influence of individual variability and the sensitivity of the method after punctual gluten exposure maintaining identical daily diet. In this study, we determined fecal GIP excretion collecting samples from all the depositions of participants under a GFD, with two separated ingestions of 50 mg and 2 g of gluten with minimal dietary variations.

## Methods

### Study design and population

**Fig. 1:**
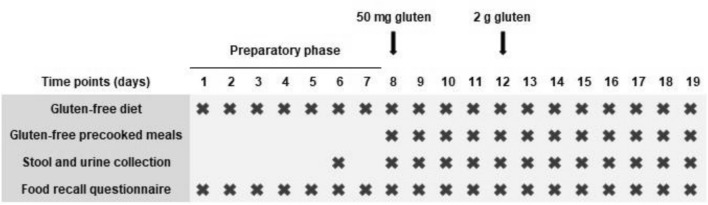
Study timeline

This prospective study included 20 healthy adults recruited between January 2020 and March 2020. The study protocol was reviewed by the ethics committee, and written informed consent was obtained from all participants (n. 2381-N-19). All participants were instructed to exclude gluten-containing food from their habitual diet for one week and to collect a urine and feces sample to confirm adherence to GFD 2 days before the ingestion of the first dose (Fig. 1). After this pre-test preparation, participants were provided with equivalent prepared gluten-free ready-to-eat lunch and dinner meals and gluten-free bread, which were supplied daily by the research team. The prepared meals were consumed within the prescribed GFD. Two doses of gluten (50 mg and 2 g) were ingested in the morning (9:00 am) on days 8 and 12, respectively, and one sample each of all the stools and urine (data published separately [33]) passed throughout the day was collected over the duration of the entire study period (12 days).

A food-recall questionnaire was used to determine GFD adherence and fluid intake from the commencement to completion of the study. Participants were instructed to record the name and the quantity of the food items that they consumed daily.

The inclusion criteria for the study were as follows: (1) age > 18 years, (2) no diagnosis of CD, non-celiac gluten sensitivity, food allergies, food intolerances, and other gastrointestinal diseases, (3) willingness to follow a strict diet and, (4) willingness to collect daily stool samples.

The exclusion criteria were as follows: (1) diagnosis of concomitant pathological conditions or severe psychiatric illnesses and, (2) inappropriately collected samples on at least 70% of all occasions.

### Gluten doses

Gluten doses were administrated in the form of gelatin capsules (“000” size, Your Supplements™, Bredbury, Stockport, England) filled with powdered wheat gluten El Granero Integral™ (Biogran S.L., Madrid, Spain). Gelatin capsules were analyzed using the GlutenTox® enzyme-linked immunosorbent assay (ELISA) Sandwich kit (Hygiena, Seville, Spain) to confirm the absence of gluten.

The gluten dosage was selected as follows: 50 mg, considered as the minimum amount of gluten that can produce histopathological changes, following daily intake in patients with CD [27], and 2 g, amount considered the dose necessary to evaluate the dynamics of fecal GIP excretion.

**Fig. 2:**
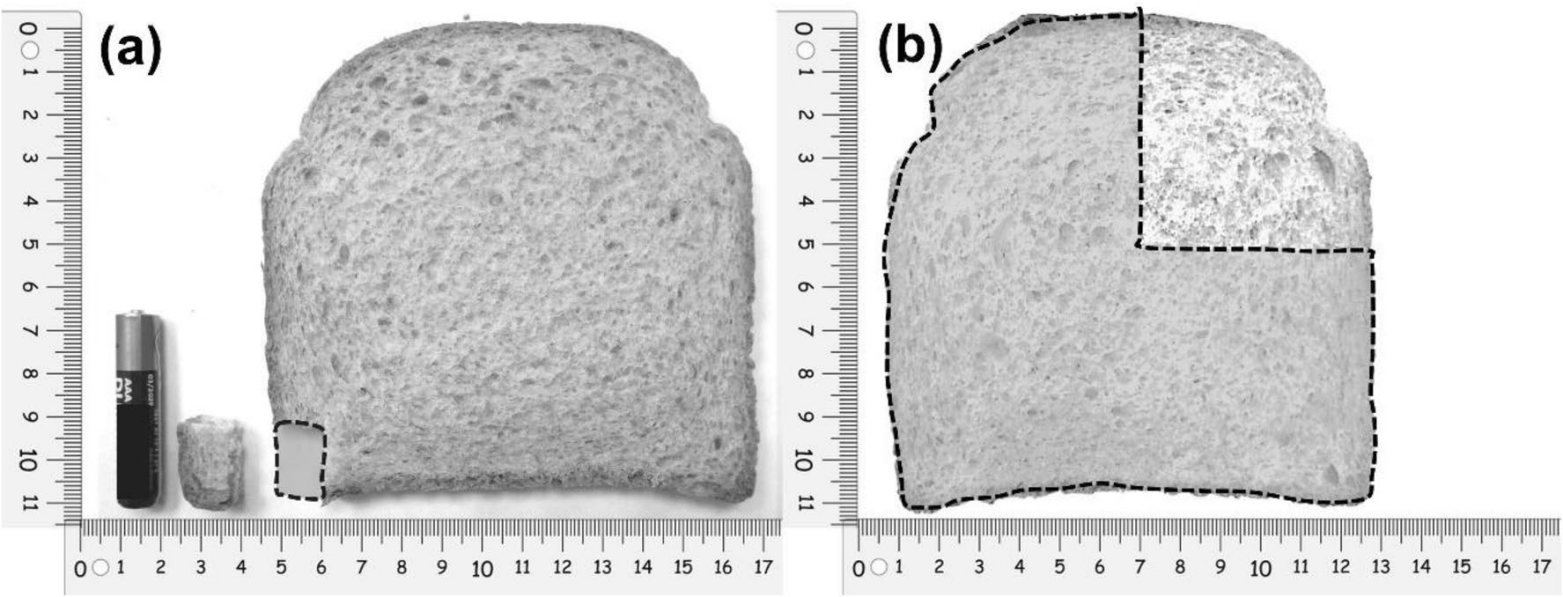
Representation of gluten doses in a slice of bread. Dashed lines show the portion of the slice which represent 50 mg of gluten (**a**) and 2 g of gluten (**b**). The small piece of bread representing 50 mg of gluten is also compared in size to a AAA battery (**a**)

We performed specific calculations using a slice of common bread based on the methodology described by Biagi et al. to interpret these gluten quantities in terms of food [34]. The slice of bread measured 11 cm × 12 cm in size and weighted 30 g. Based on the nutritional composition provided by the manufacturer, the entire slice contained 2.48 g of gluten. The corresponding amount of bread for 50 mg and 2 g of gluten were 0.6 g and 24 g, respectively (Fig. 2). A battery (AAA) was used to show the size of the pieces of bread.

Several samples of maize starch, Maizena™ (Unilever, London, England) were spiked with the powdered gluten of different concentrations and analyzed using GlutenTox® ELISA Sandwich kit (Hygiena, Seville, Spain) using G12 and A1 antibodies for gluten estimation. Based on the results obtained (nearly 100% recovery), gluten doses for each participant were prepared using the total weight of the powdered gluten as follows: 50 ± 5 mg and 2,000 ± 5 mg in 1 and 4 capsules, respectively.

### Prescribed gluten-free diet

To reduce variability in the results, all the participants followed the same isocaloric GFD prepared by a registered dietitian. Prepared ready-to-eat meals for lunch and dinner were ordered from a catering company, and all items were analyzed by the ISO17025 certified Biomedal laboratory using the GlutenTox® ELISA Sandwich kit (Hygiena, Seville, Spain) to confirm the absence of gluten. Gluten-free certified bread (Beiker™, Dr. Schär, Postal BZ, Italy) was also provided for breakfast time and to complete the meals. Fresh fruits, unprocessed nuts, and gluten-free beverage ingestion were freely permitted, depending on the energy requirements and dietary habits of each participant.

### Stool collection

Detailed instructions were provided to all the participants at the commencement of the study. Participants were provided with all the material for stool collection, including specific plastic screw-capped containers, labels, cool bags, isothermal boxes, and cool packs and were instructed to collect a minimum of 10 g of stool each time and to record the date and time of collection. All stool samples were preserved in isothermal boxes with cool packs at 4–8 °C and were submitted to the laboratory within 48 h of collection. All samples were stored at − 20 °C until they were processed.

### Stool analysis

Qualitative analysis of GIP in stool samples was performed using a lateral flow immunosorbent assay (LFIA) using the iVYCHECK GIP Stool kit (Biomedal S.L., Seville, Spain) based on the manufacturer’s guidelines. Stool samples were extracted with an ethanol–water extraction solution and shaken vigorously intermittently for 10 min. Ten drops of the extracted sample were transferred to a tube containing a dilution solution and thoroughly mixed by inverting the tube for 15 s. Thereafter, eight drops of the mixture were placed in the immunochromatographic cassette, and the results were visually interpreted after 30 min. (recommended time for samples containing a low amount of GIP). A red color at the test line and green color at the control line was interpreted as a positive result, and a green color at the control line was interpreted as a negative result. Each stool sample was tested in duplicate.

The concentration of GIP in stool samples was also measured using a sandwich ELISA technique with the iVYLISA GIP Stool kit (Biomedal S.L., Seville, Spain) based on manufacturer’s guidelines. Stool samples were incubated for 60 min at 50 °C in 5 mL ethanol–water extraction solution per mg of stool with gentle agitation to release the GIP from the stool matrix. After extraction, samples were diluted 1:10 and incubated for 60 min in the microtiter plate coated with G12 together with the standards and assay controls. The wells were subsequently washed, and the samples were incubated with horseradish peroxidase conjugated G12 antibody for the next 60 min. The plates were washed again and incubated with the horseradish peroxidase substrate. Sulfuric acid was added to prevent color development, and absorbance was measured at 450 nm using a micro-plate reader, the FLUOstar® Omega (BMG Labtech, Ortenberg, Germany) device. The range of measurement using this method was: 0.078–1.25 μg GIP/g feces. The results were expressed as μg of GIP per g feces. Each sample was run in duplicate, and at least two different aliquots of each sample were tested.

### Statistics analysis

The calculation of the sample size considered the variability found in GIP excretion between individuals under similar conditions from a pilot study performed previously at Biomedal S.L. Considering that both urine and stool GIP detection methods were studied, the most unfavorable situation was selected. The minimum expected difference was the limit of quantification (0.078), and the dropout rate was 0.2. With an alpha risk of 5%, a beta risk of 20% (80% of statistical power) and a standard deviation of 0.101 the sample size needed was 17 subjects. The calculation was made using the tool GRANMO v7.12 April 2012 (Institut Municipal d'Investigació Mèdica, Barcelona, Spain).

Quantitative variables were expressed as the mean (SD) and median (interquartile [IQR] range) and categorical variables as absolute (N) and relative (%) frequencies. The goodness-of-fit test for normality was performed using the Shapiro–Wilk test.

The Wilcoxon test was used for paired quantitative variables. Cohen’s kappa index (κ) was used to measure the degree of concordance between the evaluated investigated diagnostic techniques that showed dichotomous results, and the Landis and Koch criteria [35] were used for the interpretation of the strength of concordance.

A time range for evaluation of GIP excretion dynamic was established at intervals of 24 h, except for the first interval, which was between 0 and12 hours for better understanding of the results. All samples from each participant within each time range were clustered to obtain a single result per participant. A GIP-positive result in any of the analyzed samples was considered a positive result.

Basic probability rules were used to determine the analytical sensitivity of the investigated techniques over a predetermined time range using different samples collected for the study.

All statistical analyses and graphics were performed using IBM SPSS Statistics 25.0 for Windows (IBM Corp, Armonk, NY, United States), Epidat package, version 4.2 (Consellería de Sanidade, Xunta de Galicia, Spain, Organización Panamericana de la salud [OPS-OMS], Universidad CES, Colombia) and Graphpad Prism 9.0.2 (GraphPad Software, San Diego, CA, United States). A *P* value < 0.05 was considered statistically significant.

## Results

### Subjects and samples

**Fig. 3:**
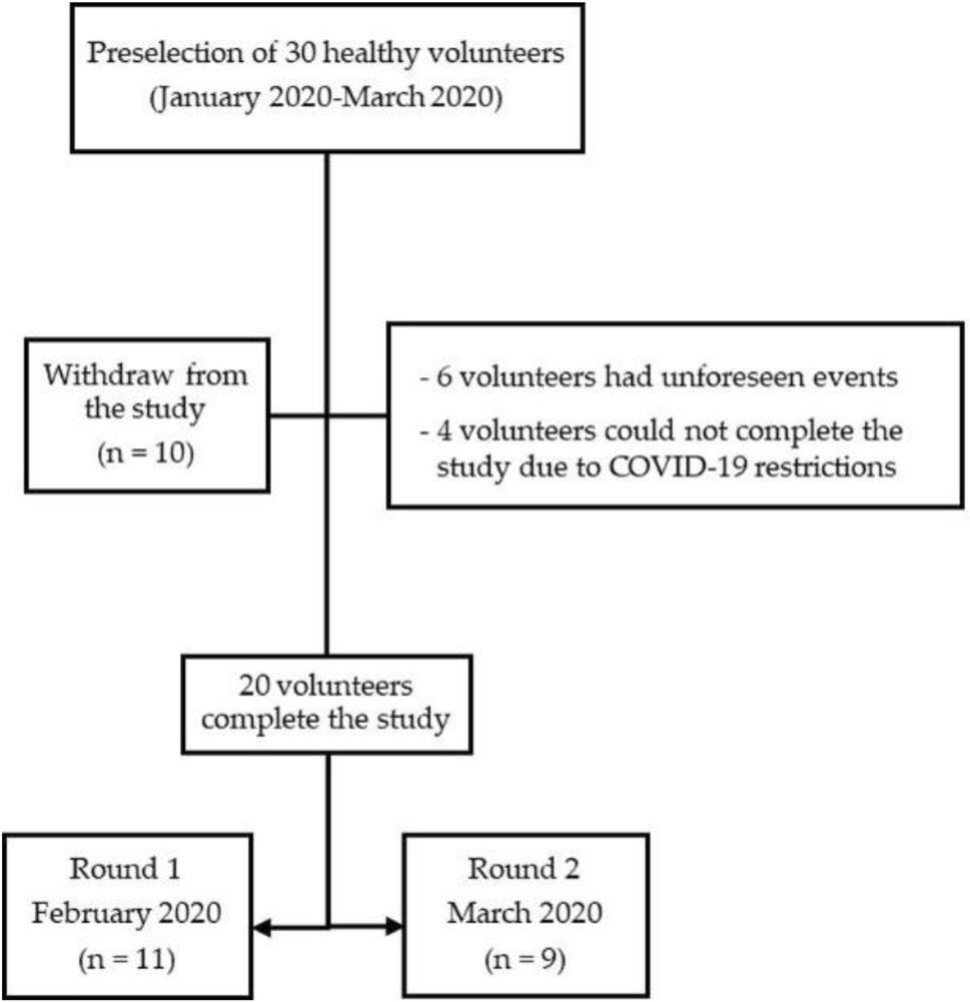
Flowchart showing distribution of the study participants

We selected 30 participants for the study between January and March of 2020. Among these, 10 withdrew from the study owing to unforeseen events (*n* = 6) and COVID-19 mobility restrictions (*n* = 4). Therefore, 20 participants, including 13 (65%) females and 7 (35%) males with a median age of 30.5 years (IQR 24.7–34 years), completed the study (Fig. 3). The study was developed in two rounds (February and March), which included 11 and 9 participants, respectively. No participants were diagnosed with a relevant disease or reported a history of probiotics/fiber supplements intake. Only one participant reported following a special fitness diet before the study.

Based on the food-recall questionnaire, all participants were compliant with the prescribed GFD and the gluten dose ingestion. The mean fluid intake per participant during the study period was 1.5 ± 0.6 L/day. The initial questionnaire that provided an overview of participants’ habits was completed by 18/20 (90%) participants. The daily meal frequency was 3 meals/day among 8/18 (44.4%) participants and 4–6 meals/day among 10/18 (55.6%) participants; lunch was the main meal of the day for all the participants. Intake of fiber-rich food varied among participants as follows: 12/18 (66.7%) participants usually had whole meal bread daily, 12/18 (66.7%) participants had ≥ 3 portions of nuts/week, 10/18 (55.6%) had ≥ 2 portions of pulses/week, 3/18 (16.7%) had < 3 portions/day, 12/18 (66.7%) had 3–4 portions/day and 3/18 (16.7%) had > 5 portions of fruits and vegetables/day. Notably, 19/20 (95%) participants participated in regular physical activity; 8/19 (42.1%) in low-intensity, and 11/19 (57.9%) in moderate-to-high intensity physical activity.

### Dynamics of fecal gluten immunogenic peptide excretion

A total of 330 fecal samples were collected from all participants; 20 from participants who confirmed GFD adherence prior to gluten ingestion, 92 corresponding to the excretion of 50 mg of gluten and 197 to the excretion of 2 g of gluten. The remaining 21 samples were excluded from the statistical analysis because GIP-positive results were obtained from the previous gluten-containing diet before study commencement. The median number of samples collected per participant was 16 (IQR 12.3–22.5), and the median stool collection frequency was 1 sample/day (IQR 1–1.5).

Stools samples analyzed over 4 days following 50 mg gluten intake showed GIP in 22/92 (23.9%) samples based on ELISA testing and in 8 (8.7%) samples based on LFIA testing, corresponding at least one of them to 13/18 (72.2%) participants and 4/18 (22.2%) participants, respectively. All samples from two participants were excluded for the 50 mg dose, because they showed GIP-positive results before the gluten intake. Stool samples analyzed over 8 days following 2 g gluten intake showed GIP in 83/197 (42.1%) samples based on ELISA testing and in 42 (21.3%) samples based on LFIA testing. At least one of the fecal samples was positive for 19/20 (95%) of participants by ELISA and 16/20 (80%) participants by LFIA, respectively (Table 1). Statistically significant differences were observed between gluten dosages regarding GIP-positive results obtained using ELISA and LFIA techniques (*P* = 0.007 and *P* = 0.003, respectively).

**Table 1:** Individual characteristics of gluten immunogenic peptides (GIP) detection in feces after 50 mg and 2 g gluten ingestions

GIP detection in feces																
50 mg gluten									2 g gluten							
Part	Sample	LFIA +	ELISA +	Time median	Time range	Peak max GIP	GIP median	GIP range	Sample	LFIA +	ELISA +	Time median	Time range	Peak max GIP	GIP median	GIP range
	*n*	*n*	*n*	*h*	*h*	*h*	*µg/g*	*µg/g*	*n*	*n*	*n*	*h*	*h*	*H*	*µg/g*	*µg/g*
1	11	1	1	47.33	–	47.33	0.08	–	13	1	6	71.88	47.33–97.75	47.33	0.11	0.09–0.33
2	6	0	0	–	–	–	–	–	12	0	2	57.88	34.25–81.50	34.25	0.18	0.17–0.19
3	5	2	2	28.50	23.50–33.50	33.50	0.17	0.07–0.26	15	8	10	65.59	23.33–183.00	32.25	0.20	0.09–0.36
4	10	3	4	52.92	34.50–64.33	51.57	0.13	0.09–0.17	13	1	1	153.00	–	153.00	0.13	–
5	6	0	1	60.00	–	60.00	0.11	–	9	4	5	71.50	30.50–125.00	54.50	0.16	0.07–0.17
6	1	0	1	31.50	–	31.50	0.10	–	4	1	1	56.25	–	56.25	0.26	–
7	7	0	1	32.50	–	32.50	0.12	–	16	6	7	31.58	12.17–104.50	12.17	0.25	0.08–0.31
8	–	–	–	–	–	–	–	–	8	1	3	68.00	20.50–117.50	68.00	0.09	0.09–0.14
9	5	0	0	–	–	–	–	–	7	0	4	132.25	23.50–169.50	169.50	0.15	0.09–0.23
10	5	0	2	34.75	11.00–58.50	11.00	0.13	0.08–0.17	16	5	8	75.88	22.83–127.50	22.83	0.17	0.09–0.32
11	4	2	4	58.17	21.83–71.25	21.83	0.18	0.11–0.27	10	2	8	94.25	22.42–167.00	46.25	0.13	0.09–0.57
12	4	0	0	–	–	–	–	–	7	1	2	89.17	73.83–104.5	104.00	0.19	0.18–0.20
13	6	0	1	25.65	–	25.65	0.08	–	14	0	8	104.92	52.40–177.50	75.75	0.12	0.08–0.28
14	6	0	1	34.43	–	34.43	0.10	–	8	2	3	48.63	23.25–73.33	23.25	0.42	0.14–0.54
16	3	0	2	38.65	27.50–49.80	49.80	0.15	0.10–0.20	7	1	2	63.99	51.17–76.80	51.17	0.18	0.12–0.24
17	5	1	1	26.00	–	26.00	0.22	–	11	2	3	34.25	28.25–51.75	28.25	0.28	0.14–0.32
19	2	0	0	–	–	–	–	–	4	0	0	–	–	–	–	–
21	4	0	0	–	–	–	–	–	9	5	6	77.21	25.75–145.42	50.92	0.28	0.09–0.62
22	3	0	1	23.33	–	23.33	0.15	–	7	1	1	25.75	–	25,75	0.19	–
23	–	–	–	–	–	–	–	–	7	1	3	73.67	30.37–98.92	73.67	0.24	0.09–0.39
TOTAL	93	9	22	34.50	11.00–71.25	32.50	0.11	0.07–0.27	197	42	83	71.88	12.17–183.00	50.92	0.16	0.07–0.62

*ELISA* enzyme-like immunosorbent assay, *GIP* gluten immunogenic peptides, *LFIA* lateral flow immunoassay, *max* maximum, *Part.* participant

GIP corresponding to the 50 mg dose were detected in only one sample in 8/18 participants (44.4%), in two samples in 3/18 participants (16.7%), and in four samples in two participants (11.1%). At the 2 g gluten dose, GIP-positive results were obtained in 1–3 samples in 8/19 participants (42.1%), 4–6 samples in 6/19 participants (31.6%), and 8–9 samples in 5/19 participants (26.3%) (Table 1). Most GIP-positive stools were obtained in the first and second samples collected after the ingestion of 50 mg of gluten by ELISA (6/18 [33.3%] and 5/17 [29.4%], respectively) and using LFIA test (2/18 [11.1%] and 2/17 [11.8%], respectively). However, among in the 2 g gluten dose, GIP-positive results were detected in the third and fourth samples collected after gluten ingestion by ELISA (12/20 [60%] and 13/20 [65%], respectively) and using the LFIA test (10/20 [50%] and 6/20 [30%], respectively).

#### Time course of gluten immunogenic peptide excretion

**Fig. 4:**
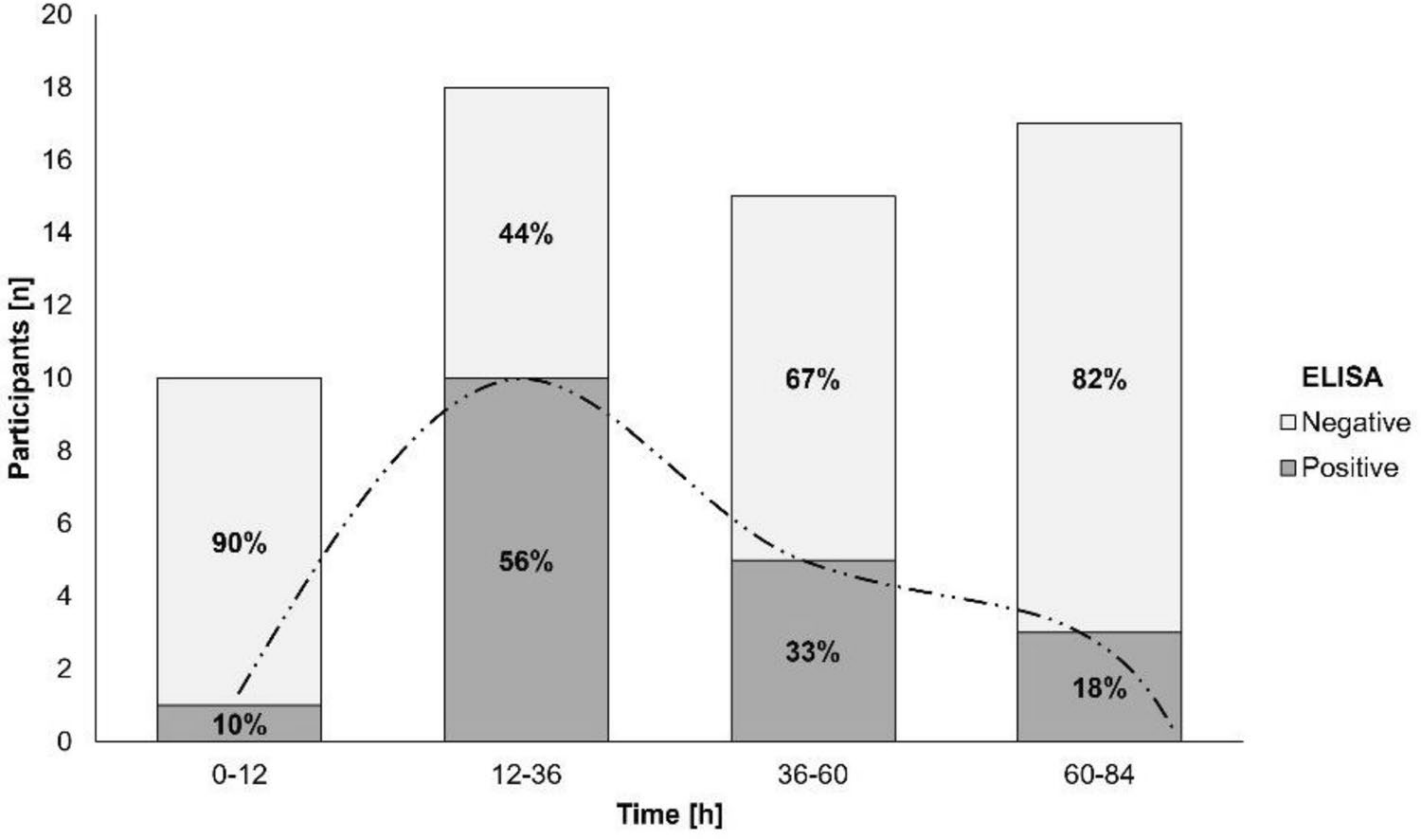
Qualitative results of fecal gluten immunogenic peptides (GIP) excretion after 50 mg of gluten intake using the ELISA. The trend of the GIP detection dynamics is represented by the dashed line

**Fig. 5:**
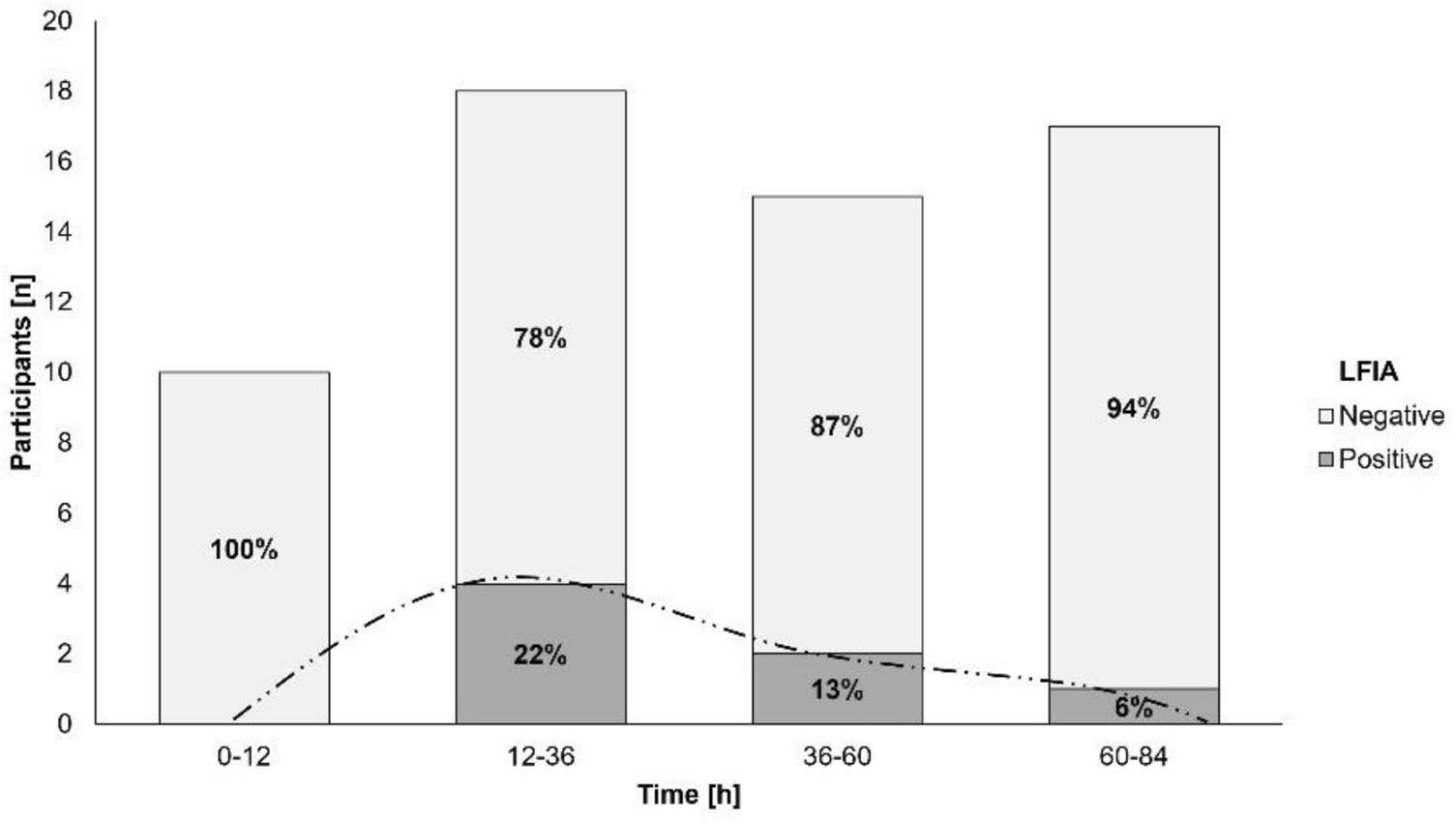
Qualitative results of fecal gluten immunogenic peptides (GIP) excretion after 50 mg of gluten intake using the lateral flow immunoassay. The trend of the GIP detection dynamics is represented by the dashed line

**Fig. 6:**
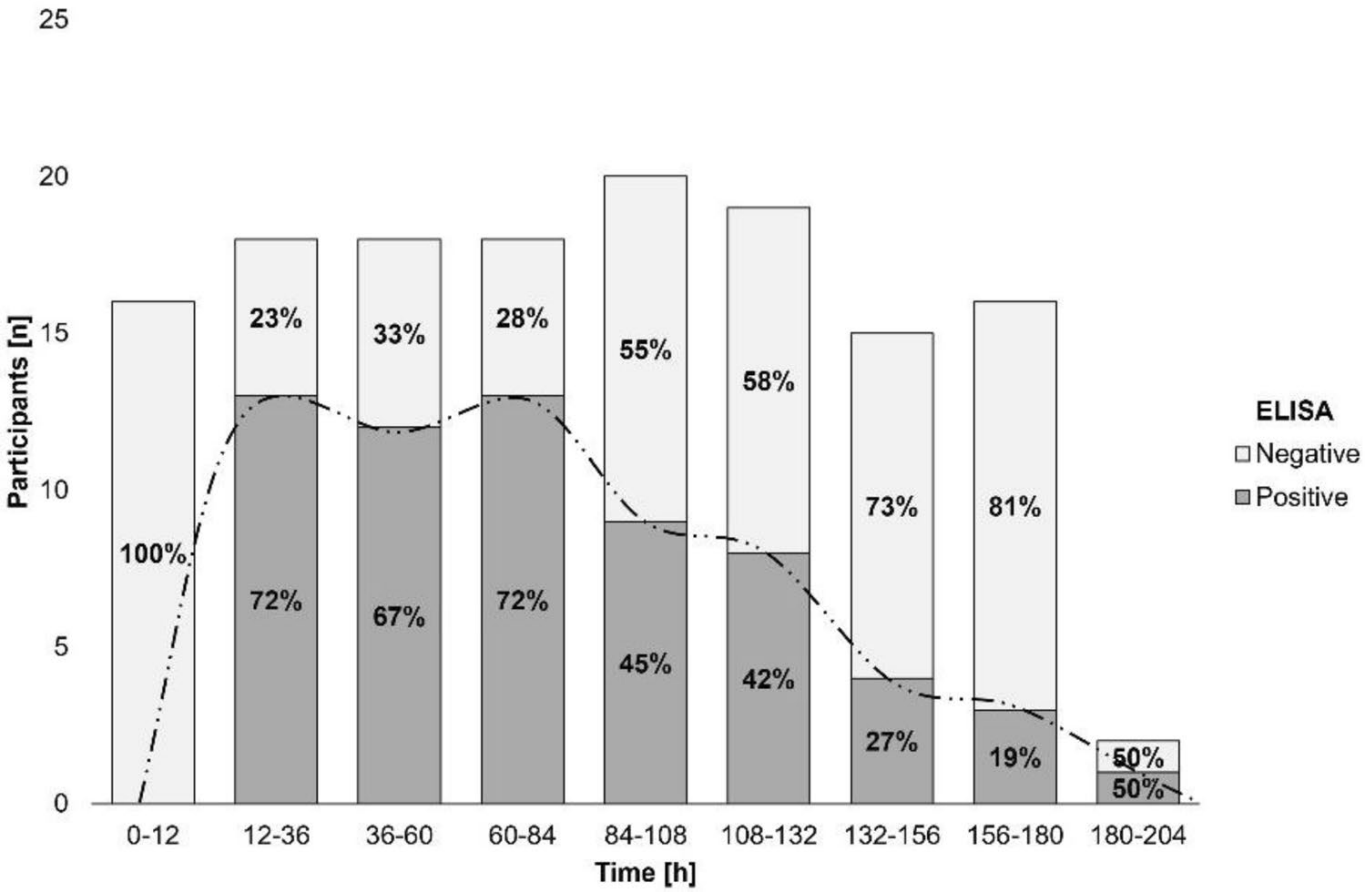
Qualitative results of fecal gluten immunogenic peptides (GIP) excretion after 2 g of gluten intake using the ELISA. The trend of the GIP detection dynamics is represented by the dashed line

**Fig. 7:**
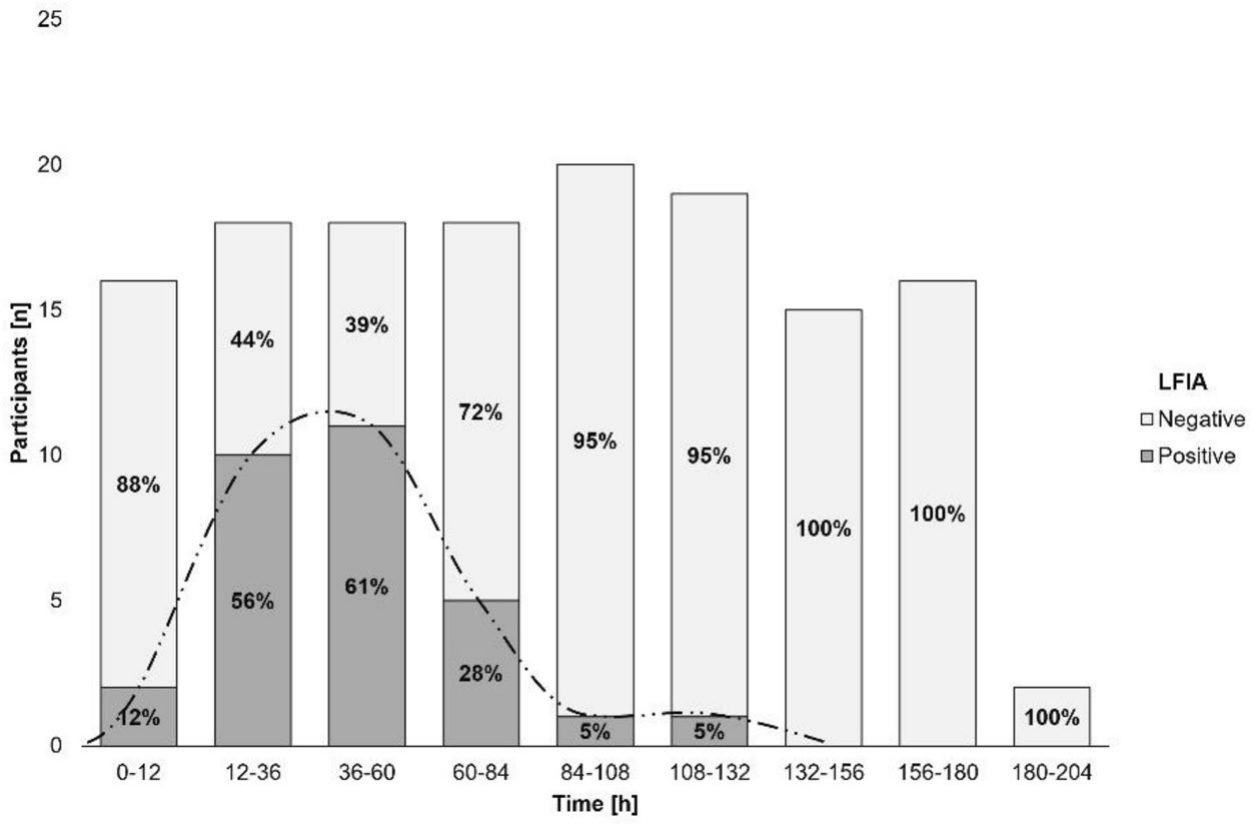
Qualitative results of fecal gluten immunogenic peptides (GIP) excretion after 2 g of gluten intake using the lateral flow immunoassay. The trend of the GIP detection dynamics is represented by the dashed line

About GIP excretion over time, most GIP-positive stools after the 50 mg gluten intake were detected between 12 and 36 h (ELISA [10/18, 55.6%] and LFIA [4/18, 22.2%] methods) (Figs. 4, 5). The 2 g dose showed similar percentages of GIP-positive stools for a longer period (12–84 h [ELISA] vs. 12–60 h [LFIA]) with rates ranging from 66.7–72.2% and 58.6–61.1%, respectively, expanding the maximum time for detection of GIP-positive stool from 72 h (50 mg) to 183 h (2 g) (Figs. 6, 7). In most participants, we observed that GIP was detected in stool samples only after a minimum duration of 20 h after gluten intake for both gluten dosages. However, GIP were detected in one participant 11 h post ingestion (50 mg dose) and in another participant after 12 h (2 g dose).

Despite significant individual variability, intake of both quantities of gluten was associated with similar median times for initial GIP detection (27.50 h [IQR 23.41–34.47]) for the 50 mg dose and 28.25 h [IQR 23.25–51.17] for the 2 g dose), and we observed not significant differences between doses (*P* = 0.239). Among the participants with GIP-positive results following ingestion of both gluten doses, 5/13 (38.5%) showed similar times regarding initial GIP detection and 8/13 (61.5%) showed differences in the first sample; 7 with differences ranging from 11–29 h and one participant with a difference 118 h in the initial sample. However, the same subject also showed discordant results regarding overall GIP detection; GIP were detected in four samples for the 50 mg dose and in only one sample for the 2 g dose.

The time range for GIP excretion differed significantly between both gluten doses, as expected; the duration was longer for the 2 g dose (0 h [IQR 0–26.06] vs. 68.55 h [IQR 25.63–104.67], *P* = 0.008). The median time for GIP detection was 34.50 h (IQR.25.91–55.25) for the 50 mg dose and 71.88 h (IQR 56.25–89.27) for the 2 g dose, and this difference between doses was statistically significant (*P* = 0.002). Most stool samples collected 72 h after ingestion of the 50 mg dose showed a GIP-negative result (last detection 71.25 h, corresponding to 3 days), whereas ingestion of the 2 g dose showed GIP-positive results until the end of the study (GIP were detectable 7 days after ingestion of 2 g gluten).

#### Concentrations of gluten immunogenic peptides in stools

Regarding GIP concentration measured by ELISA, the median among volunteers over 4 days after 50 mg ingestion was 0.02 µg GIP/g feces (IQR 0–0.06), whereas the median over 8 days after the 2 g intake was 0.07 µg GIP/g feces (IQR 0.05–0.1). Considering only those samples with a GIP-positive result the medians were 0.11 µg GIP/g feces (IQR 0.10–0.18) and 0.16 µg GIP/g feces (IQR 0.10–0.27), respectively, with significant differences between doses (*P* = 0.013).

**Fig. 8:**
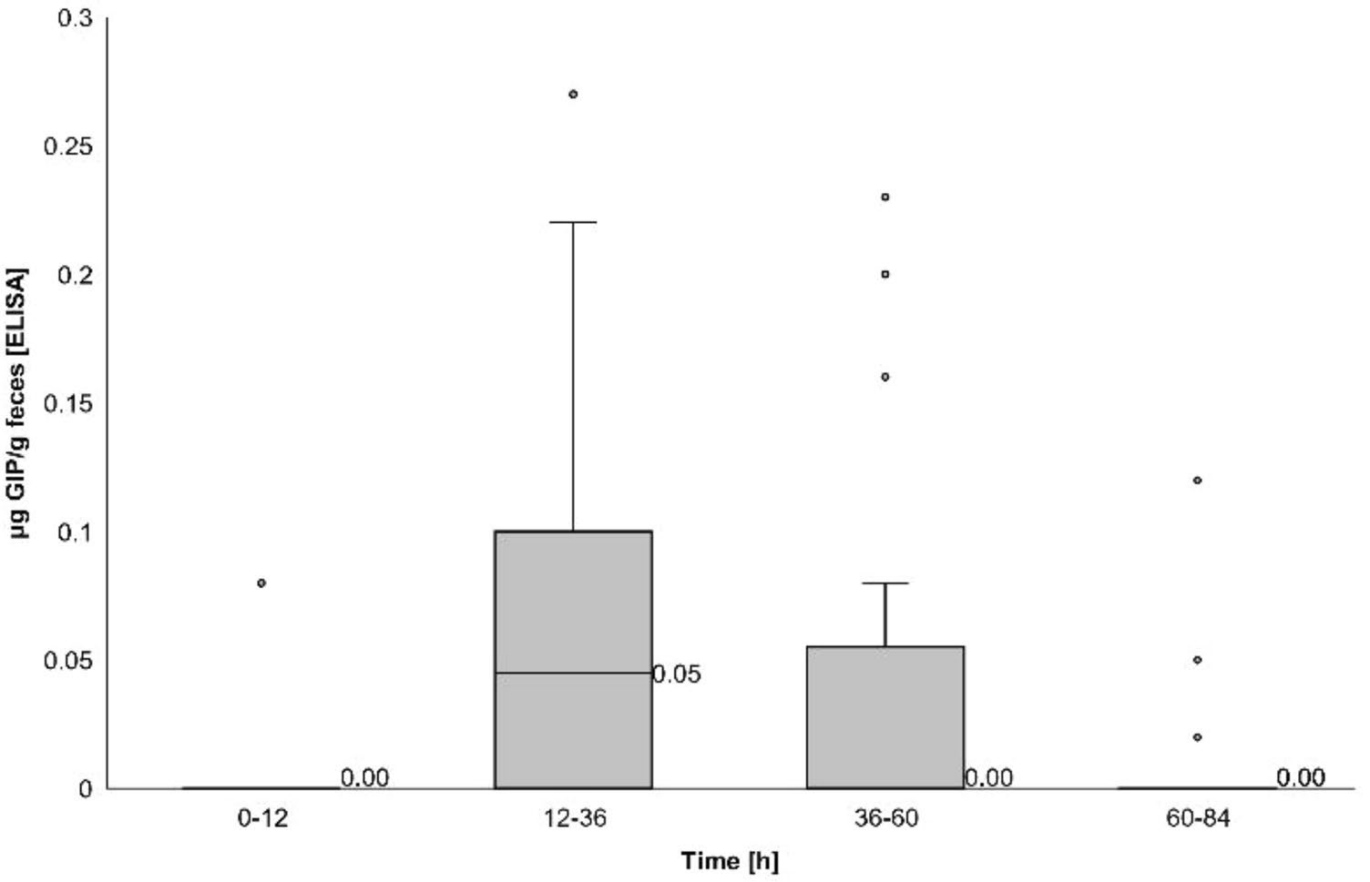
ELISA showing dynamics of fecal gluten immunogenic peptides (GIP) excretion following ingestion of 50 mg of gluten. Potential outliers are represented as dots

**Fig. 9:**
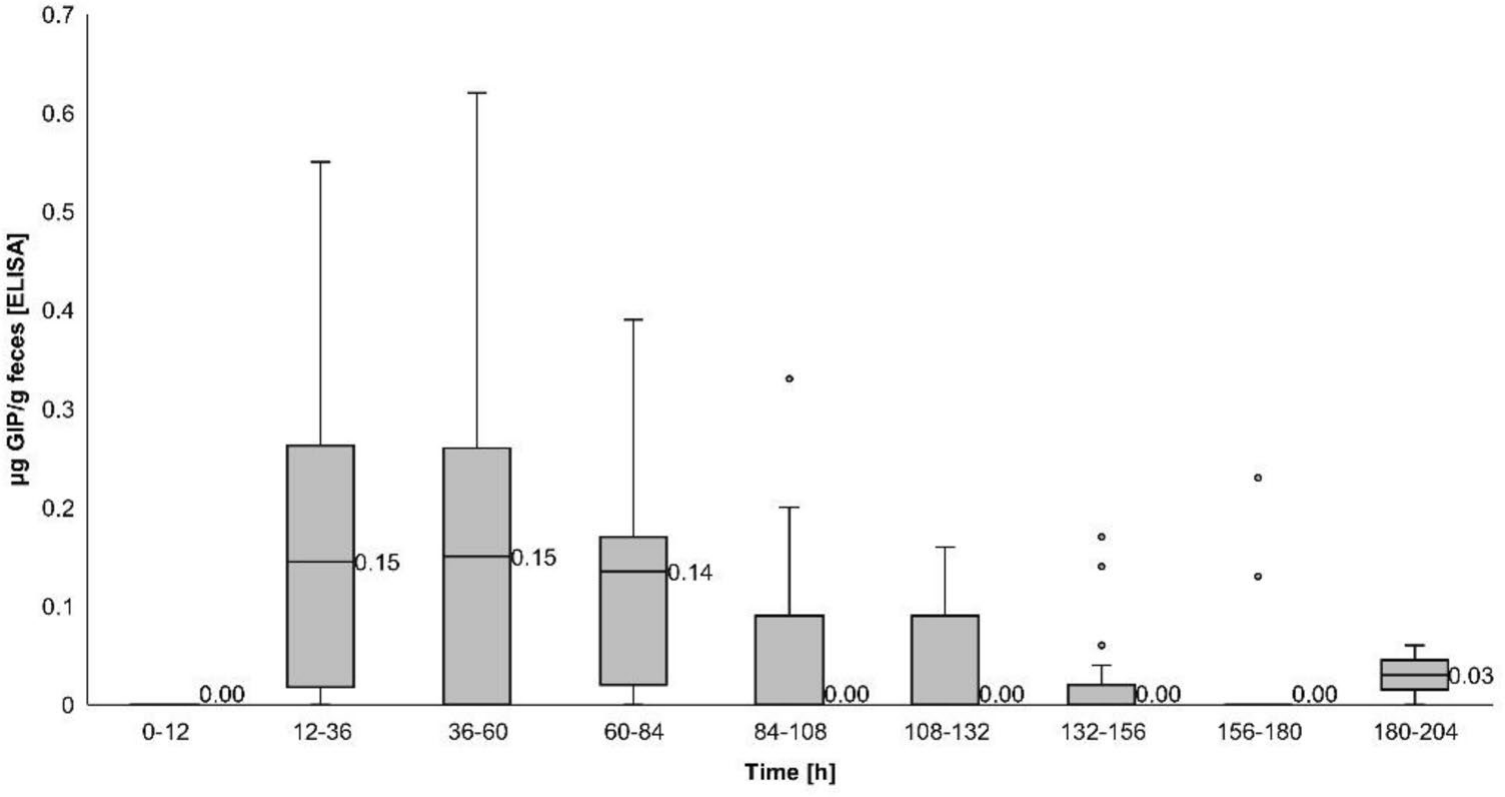
ELISA showing dynamics of fecal gluten immunogenic peptides (GIP) excretion following ingestion of 2 g of gluten. Potential outliers are represented as dots

GIP concentrations measured by ELISA were higher during the same periods, in concordance with the time required for GIP detection. The median GIP concentration in stools decreased to 0 after 36 h of ingestion of the 50 mg dose (Fig. 8, Table 2) and after 84 h of the 2 g dose (Fig. 9, Table 3).

**Table 2:** Fecal gluten immunogenic peptides (GIP) detection by ELISA in 12-h and 24-h periods after 50 mg gluten ingestion

Time	Participants	GIP + participants	GIP [µg/g]
h	n	n	Median (IQR)
0–12	10	1	0.00 (0)
12–36	18	10	0.05 (0–0.10)
36–60	15	5	0.00 (0–0.06)
60–84	17	3	0.00 (0)

*GIP* gluten immunogenic peptides

**Table 3:** Fecal gluten immunogenic peptides detection by ELISA in 12-h and 24-h periods after 2 g gluten ingestion

Time	Participants	GIP + participants	GIP [µg/g]
h	n	n	Median (IQR)
0–12	16	0	0.00 (0)
12–36	18	13	0.15 (0–0.27)
36–60	18	12	0.15 (0–0.26)
60–84	18	13	0.14 (0–0.18)
84–108	20	9	0.00 (0–0.09)
108–132	19	8	0.00 (0–0.09)
132–156	15	4	0.00 (0–0-02)
156–180	16	3	0.00 (0)
180–204	2	1	0.03 (0–0-06)

*GIP* gluten immunogenic peptides

**Fig. 10:**
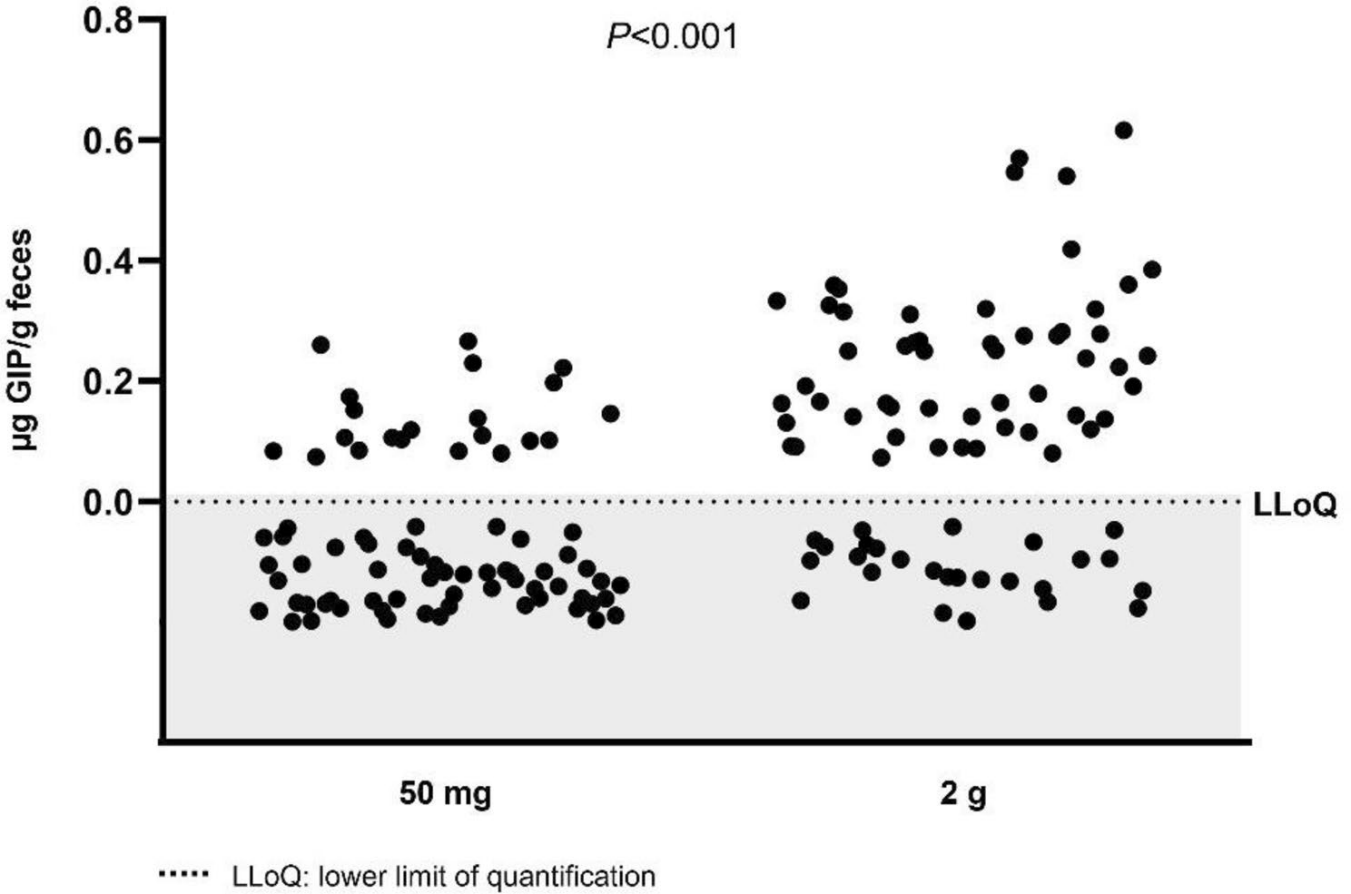
Gluten immunogenic peptides (GIP) detected in fecal samples by ELISA between 12 and 84 h after gluten ingestion of 50 mg and 2 g of gluten

Considering the range of 12–84 h after gluten ingestion, the median of GIP concentration for the dose of 50 mg was 0 µg GIP/g feces (IQR 0–0.08) and for the dose of 2 g 0.14 µg GIP/g feces (IQR 0–0.27), with statistically significant differences between doses (*P* < 0.001) (Fig. 10).

#### Individual variations in patterns of gluten immunogenic peptide excretion

**Fig. 11:**
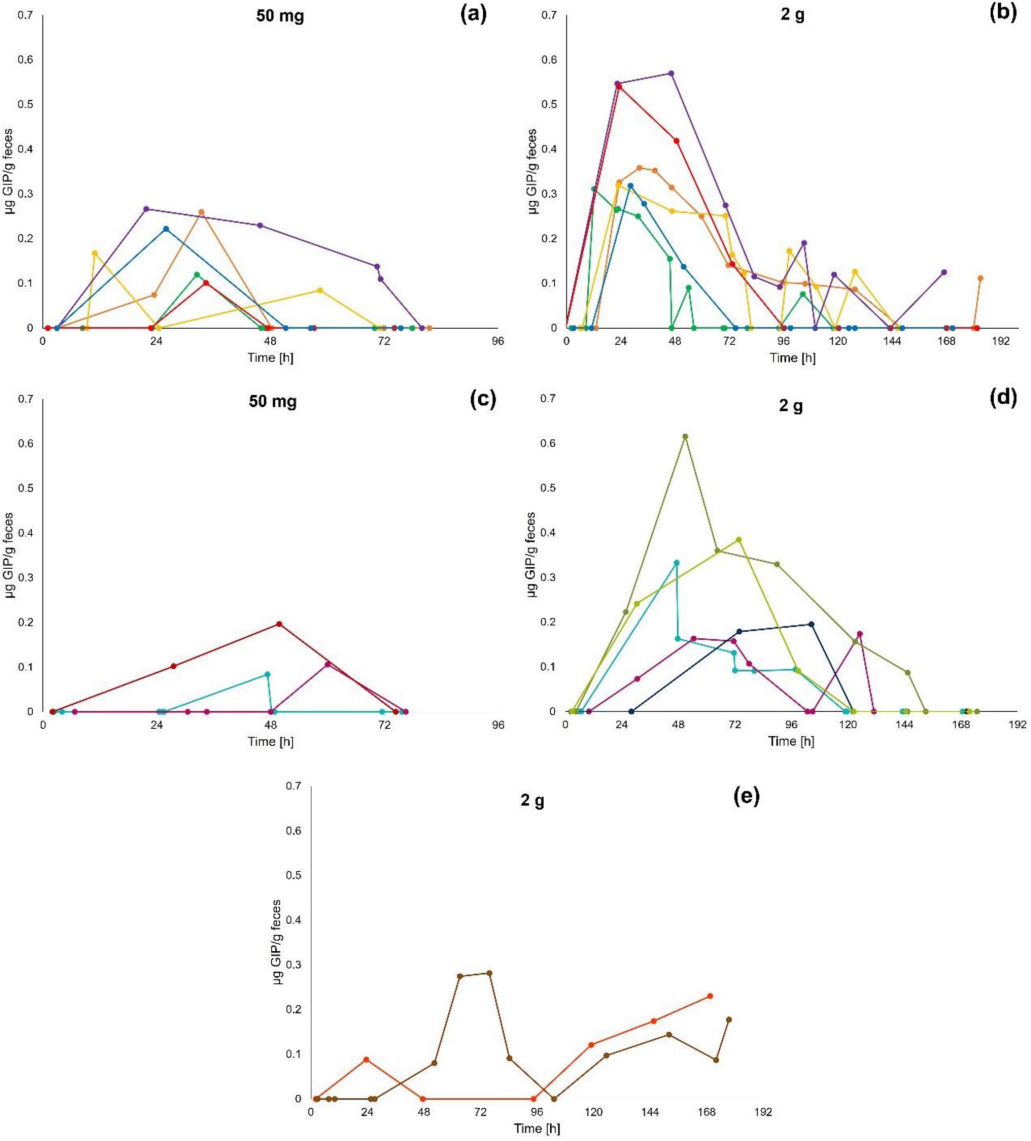
Individual variations in patterns of fecal gluten immunogenic peptides (GIP) excretion by ELISA. Peak of fecal GIP detection within 48 h after 50 mg (**a**) and 2 g (**b**) gluten ingestions, peak of fecal GIP detection from 48 h after 50 mg (**c**) and 2 g (**d**) gluten ingestions and increasing fecal GIP detection from 5 days after 2 g gluten ingestion (**e**)

Overall, we observed high interindividual variability in fecal GIP excretion with varied patterns of GIP excretion (Fig. 11), however, 9/13 subjects showed similarities in excretion patterns with both doses of gluten (Table 1). Some subjects showed peak fecal GIP concentrations within the first 48 h after ingestion with reduced gluten elimination in the consecutive samples over the study period (Fig. 11a, b). Six of these participants showed a similar pattern with both 50 mg and 2 g gluten intakes but three of them showed higher GIP concentrations after the 2 g dose. Furthermore, other participants showed peak GIP concentration after 48 h, three of these with similar times for both gluten ingestions and with discordant results observed in one subject (Fig. 11c, d). Interestingly, two different participants showed an increase in the fecal GIP concentration at the end of the study, but only after ingestion of 2 g of gluten (Fig. 11e). Additionally, GIP concentrations over and under the limit of detection were observed in many participants during the study period (Fig. 11b, d, e).

When the results were compared between sex, higher GIP concentrations were seen in the group of males in 2 g gluten intake; however, no statistical significance was observed between females and males in GIP detection (0.14 µg/g vs. 0.12 µg/g, respectively, for the 50 mg dose (*P* = 0.734); 0.17 µg/g vs. 0.29 µg/g, respectively, for the 2 g dose (*P* = 0.173)). Furthermore, when 50 mg of gluten where ingested similar results were found between females and males in terms of initial time of GIP detection ((30.88 h vs. 30.09 h, respectively, (*P* = 0.866)) and final time of GIP detection (46.79 h vs. 30.09 h, respectively, (*P* = 0.176)). Despite females showed a larger time for initial and final GIP detection than males in the 2 g gluten intake, no statistically significant differences were found (48.75 h vs. 20.39 h, respectively (*P* = 0.136) for initial GIP detection and 116.01 h vs. 67.86 h, respectively (*P* = 0.380) for final GIP detection).

#### Diagnostic sensitivity of analytical methods

GIP detection capacity was higher even for a smaller gluten dose with the ELISA than with the LFIA test. Fecal GIP were detected in at least one sample in 13/18 (72.2%) participants after 50 mg gluten intake within a time interval of 12–84 h, and this rate increased to 18/20 (90%) following intake of 2 g of gluten. In contrast, the LFIA method was less sensitive and detected GIP in 4/18 (22.2%) subjects after 50 mg gluten ingestion and in 15/20 (75%) subjects after 2 g gluten ingestion during the same period (12–84 h). Most of GIP-positive samples detected using LFIA also showed positive results using the ELISA method, but we observed discordant results between methods in six samples even after analysis of three aliquots with ELISA and two aliquots with LFIA. However, due to the methods have different sample extraction protocols any aliquot was analyzed with both tests. In all discordant cases the intensity of the test line was very light, which indicates that the GIP concentration of the sample was close to the limit of detection. Moreover, most GIP-negative results based on LFIA testing (which were positive using ELISA) were in the range of 0.08–0.15 µg GIP/g feces, concentrations below the limit of detection of the LFIA test. Based on these data, comparison between both methods revealed slight concordance about the 50 mg dose (Cohen kappa 0.35, 95% confidence interval [CI] 0.14–0.56, *P* < 0.001), which high to moderate concordance in the 2 g dose, following comparison of all results obtained from both methods (Cohen kappa 0.43, 95% CI 0.31–0.55, P < 0.001; Cohen kappa 0.42; 95% CI 0.32–0.53, P < 0.001, respectively).

Considering 12–84 h after gluten intake as the interval of time suited for GIP detection using both techniques, we calculated the theoretical probability of at least one GIP-positive stool sample after a single gluten ingestion (Table 4). The diagnostic sensitivity of the ELISA test for detection of GIP in a sample after digestion of a small amount of gluten (50 mg) was 27.3%, which increased to 47.1% in two and to 61.5% in three samples. Although the LFIA test showed a lower sensitivity for the same quantity of gluten intake in one sample (10.4%), the probability reached 19.7% in two and 28% in three samples.

**Table 4:** Comparison of diagnostic sensitivity of the ELISA and lateral flow immunoassay within a specific time range and across different samples

	Sensitivity													
		1 sample				2 samples				3 samples				
	Time [h]	***12–84***	12–36	36–60	60–84	***12–84***	12–36	36–60	60–84	***12–84***	12–36	36–60	60–84	
50 mg gluten	LFIA [%]	***10.4***	14.8	12.5	3.9	***19.7***	27.4	23.4	7.5	***28.0***	38.2	33	11.1	
	ELISA [%]	***27.3***	40.7	25	15.4	***47.1***	64.9	43.8	28.4	***61.5***	79.2	57.8	39.4	
2 g gluten	LFIA [%]	***46.3***	59.3	54.2	27.6	***71.1***	83.4	79	47.6	***84.5***	93.2	90.4	62	
	ELISA [%]	***67.5***	66.7	66.7	69	***89.4***	88.9	88.9	90.4	***96.6***	96.3	96.3	97	

Interval of time convenient for GIP detection is in bolditalics

*LFIA* lateral flow immunoassay, *ELISA* enzyme-like immunosorbent assay

We observed that the sensitivity of a single sample could be as high as 67.5% using ELISA, and 46.3% using LFIA, following the daily ingestion of a significant amount of gluten (2 g); rates could be as high as 89.4% and 96.6% with the ELISA test and 71.1% and 84.5% with the LFIA test in two and three samples, respectively.

## Discussion

In this article, we describe the dynamics of human fecal GIP excretion in conditions simulating occasional gluten exposure under controlled dietary conditions to study individual variability. This is the first study collecting samples of all the following defecations after gluten intake and controlling diet variables to determine the range of individual variability in the excretion of fecal GIP.

In this study, ingestion of a single dose of 50 mg of gluten was associated with GIP-positive stool over a period of 11–72 h in healthy subjects with a median time of 27 h required for the initial detection and a peak of detection from 12 to 60 h, with most GIP excreted in only a single sample per participant. Moreover, ingestion of a 40-fold higher dose of gluten (2 g), was associated with similar results regarding the time interval after which most samples showed GIP-positive results (12–84 h), and no statistically significant differences were observed regarding the initial time of GIP detection (27 h vs. 28 h, *P* = 0.239). However, the time range for GIP detection significantly differed depending upon the amounts of gluten ingested. GIP remained in the gastrointestinal tract of participants for a median time of 72 h; however, the duration of GIP detection was as long as 7 days in a few individuals. It is possible that undetectable GIP could also get eliminated during that period after ingestion of 50 mg of gluten in some participants, but the amount of GIP may not reach the threshold level considering the sensitivity of methods used in the study.

Our results agreed to those reported by other studies. Comino et al. [17] first reported that the ELISA method based on anti-33mer monoclonal antibodies could detect gluten-derived peptides in the feces of patients with CD and healthy volunteers, following ingestion of processed bread containing between 50 mg and 30 g of gluten. These authors observed that the time required for gluten-derived peptide excretion was from 2–4 days in six volunteers. Silvester et al. observed similar findings in patients with CD in whom they confirmed an association between definite gluten exposure and GIP positivity in stools 2–4 days after gluten ingestion [32]. However, it was not fully feasible to accurately establish the expected period of detection owing to the high interindividual variability observed in these studies. Our results obtained in a larger number of participants (*n* = 20) showed GIP detection between the 2nd and 7th days of a gluten challenge.

A study performed in a small group of healthy participants observed the kinetics of fecal GIP elimination over a week after the start of a GFD, and the authors found high interindividual variability; detection of GIP occurred over > 3 days in some and even until the end of the week in other participants [30]. Although the specific moment and quantity of gluten ingestion were not reported, the results of our study were consistent with those of the aforementioned study with regard to the diversity in GIP elimination patterns. In fact, all the examples described in this publication were represented by the participants in our study: however, our results revealed that the individual GIP excretion pattern may be independent of the amount of gluten ingested, considering the initial time of detection and peak of maximum excreted GIP. The amount of GIP is expected to decrease over time for a single gluten dose; however, interestingly, in both studies, we observed intermittent GIP-positive and GIP-negative samples. Furthermore, in two participants a significantly higher fecal GIP content was observed after several days without any fecal GIP detection. The eventual ingestion of gluten was unlikely because parallel urine test controls were negative. These findings may be attributable to the discrete gluten particles that could remain temporarily encapsulated by food matrixes or occasionally retained in the intestine. Although this phenomenon only occurred after the ingestion of 2 g of gluten, it cannot be ruled out that this pattern was also present in the 50 mg dose, but perhaps GIP were undetectable because the amount of gluten was 40-fold lower.

Roca et al. [31] investigated the dynamics of GIP clearance in stools over 6 days in 18 recently diagnosed pediatric patients with CD, who were provided a GFD, and observed that GIP decreased over time in a non-linear manner; however, GIP recovery decreased in the first 48 h, with minimal detection in most samples between 48 and 72 h. Considering the clearance time for quantities of gluten ranging from 0.7 g to 11.6 g/day used in this study, we observed discrepancies in our results; we could expect at least minimal GIP excretion at 72 h. However, this discordance could be attributed to possible inaccuracies in the food-recall questionnaire used for gluten intake estimation.

With regard to the fecal GIP concentration, previous studies have described a weak association between the amount of gluten ingested and fecal GIP excretion [17, 31, 36, 37]. Our results confirmed this association; we observed significant differences in fecal GIP content between volunteers who were administered 50 mg and 2 g gluten (*P* = 0.013) with a significantly greater variation when results were determined in the 12–84 h range (*P* < 0.001). However, as expected, we observed high interindividual variability, with median GIP ranging from 0.08 µg/g–0.22 µg/g for the 50 mg dose and 0.09 µg/g–0.42 µg/g for the 2 g dose. The aforementioned studies reported fecal GIP quantities of 0.4 µg GIP/g feces–7 µg GIP/g feces with daily gluten ingestion ranging from 50 mg to 1 g in patients with CD and 0.2 µg GIP/g feces–29 µg GIP/g feces with a normal GCD in healthy subjects [17]. Roca et al. [37] also reported a mean of 13 μg GIP/g feces (range 0.56 μg/g–47 μg/g) following gluten intake ranging from 0.5 g/day to 10.5 g/day. The disparity between our results and those of previous studies could be attributed to the matrix containing gluten used in the study (capsule with partially purified gluten in this study vs. normal food in previous studies), the methodology used for the estimation of gluten ingestion, and the frequency of gluten ingestion.

In this study, we confirmed the sensitivity of the ELISA method for frequent detection of gluten ingestion of 50 mg, which concurs with results of a previous study [17]. Although the ELISA method showed higher sensitivity than the LFIA test, both techniques showed concordance. Similar results were reported by other studies [17, 36, 37], in which diagnostic sensitivity of the ELISA method ranged from 98.5 to 100% with diagnostic specificity of 100%, and the diagnostic sensitivity and specificity of the LFIA method were 75% and 100%, respectively, showing moderate concordance [17, 37]. False-positive results were discarded in our study because the extended experience in the specificity of the G12 immunomethods either in food or in human samples. According to this assumption, GIP detection was observed after gluten ingestion and subsequently to consecutive GIP-negative samples that confirmed the compliance of the volunteers performing the GFD. Samples from those participants with moderate levels of GIP in feces after the wash-out week were excluded as we could not assure that GIP detection was from the 50 mg gluten dose. Previous studies with volunteers undergoing a GFD showed absence or very low rate of positive excreted GIP [17, 37]. Moreover, we have a rigorous control of the gluten content of the food consumed by the participants (see above in “Materials and methods”).

Discordant results could be explained by differences in the configuration of immunomethods (A1/G12 antibodies for the LFIA test and G12/G12 for the ELISA test) and the different efficiency of GIP extraction methods. Usually, samples with GIP-negative result on ELISA testing also show negative results with the LFIA test; but some samples that show a GIP-positive result by ELISA could have a low GIP content that is below to the limit of detection of the LFIA test [22, 31, 37]. However, some discrepancies may occur; for example, we observed six GIP-positive results using LFIA, although these samples tested negative using ELISA testing. This difference may be associated with the heterogeneous distribution of GIP in feces, or the antibody pair used in each method, which may show differing affinity for some peptides, as mentioned earlier. The large heterogeneity of feces composition, even in the same sample of the same individual was already described by other authors [38–40]. In this study, we aimed to reduce such heterogeneity by the homogenization of each fecal sample prior to analysis and by the determination of GIP from different aliquots taken from the same sample from each participant with each method. Nonetheless, as LFIA and ELISA tests have different extraction protocols for feces samples, each aliquot was analyzed independently with both tests.

Our results can serve to design the recommendations for the sampling of the immunotechniques to increase the sensitivity and estimate the source of GFD transgressions. Reportedly, patients with CD tend to show frequent dietary transgressions despite efforts to strictly follow a GFD [17, 22, 24, 32]; however, inadvertent gluten intake is difficult to determine. Gluten intake in individuals who are prescribed a GFD, could be secondary to regular dietary non-compliance or inadvertent transgressions. Detection of fecal GIP could likely indicate gluten intake in the preceding 12–72 h. An increase in the frequency of stool tests appears to be a convenient approach to avoid false-negative results in those showing non-compliance with GFD resultant from occasional gluten intake [22, 31]. Stefanolo et al. [22] reported that 62% of patients showed at least one GIP-positive result in weekly stool samples obtained over a month, with a median of three positive results during this period. Considering that minimal daily gluten ingestion (50 mg) can cause mucosal injury in most patients with CD and being it useful to adopt a more realistic approach with regard to a GFD, it may be convenient the use of several fecal GIP tests for a week and separated 3–4 days to include weekdays and weekends (for example, Tuesday/Wednesday and Friday/Saturday). The increase of frequency of stool sample collection could solve any clinical need to improve sensitivity of the method for detection of low-single gluten intakes. For instance, the collection of three stool samples to cover gluten exposure on both weekdays and weekends may increase the diagnostic sensitivity of the ELISA to 61.5% for 50 mg of gluten intake and 96.6% for 2 g gluten ingestion.

Overall, our study highlighted high interindividual variability in excretion time and GIP concentrations, which concurs with the results of previous research in this domain. The heterogeneity observed could be attributed to multiple contributors including gastrointestinal transit time, intestinal permeability, hydrolytic capacity of human enzymes, and intestinal microbiota activity. Several studies have reported that both patients with CD and healthy subjects have gluten-degrading bacteria that can hydrolyze immunogenic peptides and fecal glutenasic activity in individuals is inversely associated with the amount of gluten excreted [15–17, 41, 42]. These factors may explain the negative results in all the stool samples collected after 50 mg and 2 g gluten ingestions despite positive results for urinary GIP after the second dose in one participant in this study [33]. However, the possibility of a missing sample in this participant might not be discarded. In our study, we controlled the gluten dosage and dietary composition to minimize the variables analyzed. The significant differences among participants highlight the realistic scenario that should be considered when establishing protocols for stool collection for GFD monitoring.

In the present study, the variability found between participants in the detection of GIP in urine [33] and feces makes difficult to establish a general pattern between the individual excretion of GIP in both types of samples. Apparently, some of the factors previously mentioned might affect GIP excretion in urine and feces in like manner, but we observed that elements such as fluid intake may significantly modify the sensitivity of the GIP test in urine samples [33], independently of the individual. Despite the lack of strict associations between both type of samples, it was possible to observe detectable amounts of GIP in almost all individuals after the two gluten intakes in either urine or feces (only 4 participants have negative results in both samples after the 50 mg dosage). Besides, we perceived some coincidences in the detection of GIP in urine and feces in several participants. For instance, considering the initial time of GIP detection after 2 g gluten intake, 4 participants were the first ones to excrete detectable amounts of GIP in urine (3–5 h) and feces (22–25 h), and 2 participants had a delayed detection of GIP (8 h and 51–52 h, respectively). In addition, 3 participants showed low median concentrations of GIP in both types of samples after the 2 g ingestion and, on the contrary, the urine and feces samples from one participant were within the highest GIP concentration medians.

Immunoassays for fecal GIP detection are useful in patients diagnosed with CD and gluten-related disorders. A limitation of this study was that only healthy volunteers were included, which was mainly due to ethical issues. There is no sufficient evidences that the metabolism of gluten proteins is different between patients with CD and healthy individuals; however, previous research has reported that patients with CD could show digestive abnormalities in the digestion process [43]. It has also been reported that intestinal microbiota involved in gluten metabolism could be altered in patients with CD [41]. Future studies in these patient populations may confirm the equivalences and any potential deviations in the dynamics of gluten excretion compared to healthy population. Keeping in mind this concern, this report may be valuable to define the protocols for the application of fecal GIP estimation to assess gluten exposure during follow-up of gluten-induced disorders in real-world clinical practice.

## Declarations

### Availability of data and material

Not registered.
